# Weight Loss as a Determinant of Histological Improvement in Metabolic Dysfunction‐Associated Steatotic Liver Disease in People With Obesity. A Systematic Review and Network Meta‐Analysis of Randomised Clinical Trials

**DOI:** 10.1111/dom.70617

**Published:** 2026-03-09

**Authors:** Matteo Monami, Amanda Belluzzi, Silvio Buscemi, Luca Busetto, Ricardo Cohen, Maurizio De Luca, Andrea Galli, Edoardo Mannucci, Tarissa Z. Petry, Benedetta Ragghianti, Paolo Sbraccia, Dror Dicker

**Affiliations:** ^1^ Careggi Teaching Hospital and University of Florence Florence Italy; ^2^ Rovigo Hospital, ULSS5 Polesana Rovigo Italy; ^3^ Department of Promozione della Salute Materno‐Infantile, Medicina Interna e Specialistica di Eccellenza (PROMISE), University of Palermo Palermo Italy; ^4^ Department of Medicine University of Padova Padova Italy; ^5^ The Center for Obesity and Diabetes, Hospital Alemao Oswaldo Cruz Sao Paulo Brazil; ^6^ Department of Systems Medicine University of Rome Tor Vergata Rome Italy; ^7^ Internal Medicine D and Obesity Clinic, Hasharon Hospital‐Rabin Medical Center, Faculty of Medicine, Tel‐Aviv University Tel‐Aviv Israel

**Keywords:** endoscopic bariatric procedures, metabolic bariatric surgery, metabolic dysfunction‐associated steatotic liver disease, network meta‐analysis, obesity, obesity management medications

## Abstract

**Background:**

Metabolic dysfunction‐associated steatotic liver disease (MASLD) is closely linked to obesity and insulin resistance, and sustained weight loss is associated with histological improvement. Whether different obesity‐management modalities exert weight‐independent hepatic effects remains uncertain.

**Methods:**

We conducted a systematic review and network meta‐analysis (NMA) of randomised controlled trials evaluating lifestyle intervention, obesity management medications, endoscopic sleeve gastroplasty and metabolic and bariatric surgery in adults with BMI ≥ 27 kg/m^2^ and biopsy‐confirmed MASH. The primary endpoint was MASH resolution without worsening of fibrosis. Study‐level meta‐regressions explored associations between total body weight loss (TBWL%) and histologic outcomes.

**Results:**

Six RCTs (*n* = 1379) met inclusion criteria. Tirzepatide, semaglutide, sleeve gastrectomy and Roux‐en‐Y gastric bypass were superior to placebo or standard care for achieving MASH resolution. Because the network was weakly connected and largely placebo‐anchored, indirect estimates were imprecise. Across study arms, greater TBWL% was associated with higher rates of MASH resolution and fibrosis improvement; however, these associations were strongly influenced by a small number of high‐weight‐loss surgical arms.

**Conclusions:**

Weight loss was consistently associated with histologic improvement across available RCTs. However, the limited evidence base, sparse network structure and ecological nature of the meta‐regression preclude causal inference. These findings should be considered exploratory and hypothesis‐generating, underscoring the need for adequately powered head‐to‐head trials.

## 1 Introduction

Metabolic dysfunction‐associated steatotic liver disease (MASLD) is now the most prevalent chronic liver disease worldwide, affecting roughly one‐third of adults and imposing substantial clinical and economic burdens [1, 2, 3, 4, 5, 6]. The updated nomenclature underscores the central role of metabolic dysfunction—particularly obesity, insulin resistance and Type 2 diabetes mellitus (T2DM)—in disease development. Obesity contributes to MASLD through increased hepatic exposure to free fatty acids, insulin‐driven de novo lipogenesis, altered adipokine signalling and chronic low‐grade inflammation, which together promote progression from steatosis to metabolic dysfunction‐associated steatohepatitis (MASH) and fibrosis [7, 8].

Given this strong pathophysiological link, weight reduction remains a key therapeutic target: sustained losses of 7%–10% of body weight are associated with meaningful improvements in steatosis, inflammation and even fibrosis [9, 10, 11]. Multiple interventions can achieve clinically significant and durable weight loss, including structured lifestyle interventions (LSI), obesity management medications (OMMs) such as GLP‐1 receptor agonists and dual GLP‐1/GIP agonists, endoscopic sleeve gastroplasty (ESG) and metabolic bariatric surgery (MBS) [12, 13, 14, 15]. Although these modalities differ markedly in mechanism, durability and magnitude of weight loss, their relative effects on histologic MASH outcomes remain incompletely defined. Furthermore, whether hepatic improvements arise solely from weight loss or also reflect weight‐independent biological actions is uncertain.

To address these gaps, we conducted a systematic review and network meta‐analysis (NMA) restricted to randomised controlled trials (RCTs) with biopsy‐confirmed MASH and blinded histologic assessment. Our objectives were to compare the effects of established weight‐loss interventions on biopsy‐based endpoints and to explore associations between total body weight loss (TBWL) and histologic improvement using study‐level meta‐regression.

## 2 Materials and Methods

### 2.1 Reporting and Study Design

This systematic review and NMA was conducted in accordance with the Preferred Reporting Items for Systematic Reviews and Meta‐Analyses (PRISMA) extension statement [16] for NMAs (PRISMA‐NMA; Table 1S). The protocol for this meta‐analysis (MA) and NMA was pre‐registered on the PROSPERO website (https://www.crd.york.ac.uk/prospero/#recordDetails, registration number: CRD420251148787), without post hoc modifications to eligibility criteria, interventions, or outcomes.

### 2.2 Search Strategy and Selection Criteria

RCTs that enrolled patients with a body mass index (BMI) greater than 27 kg/m^2^ and steatohepatitis were included. These trials compared LSI (based on Medical Nutrition Therapy [MNT], including any low‐calorie diets [LCD] or very low‐calorie diets [VLCD] and structured physical activity programs), OMM, EBP and MBS against placebo/standard care (Pbo/SoC) or compared two active treatments. Eligible trials were required to have a minimum follow‐up of 26 weeks. This review intentionally restricted the analysis to biopsy‐confirmed endpoints to ensure the highest diagnostic rigour. No language or date restrictions were applied.

A comprehensive literature search was conducted in Medline, Embase and the Cochrane Central Register of Controlled Trials (CENTRAL) up until November 1, 2025. The full search strategy and keywords used are detailed in Table 2S. Duplicate records were removed using EndNote X9 (Clarivate Analytics, Philadelphia, PA, USA). Paired reviewers (B.R. and A.B.) independently screened titles, abstracts and full‐text manuscripts, extracting relevant data from studies meeting the inclusion and exclusion criteria.

### 2.3 Interventions

- LSI: Structured programs incorporating MNT (including LCD or VLCD) and supervised or prescribed physical activity.
- OMMs: Orlistat (360 mg), naltrexone/bupropion (32/360 mg), liraglutide (3.0 mg), semaglutide (2.4 mg) and tirzepatide (10–15 mg).
- Endoscopic bariatric procedure (EBP): ESG.
- MBS: Sleeve gastrectomy (SG) and Roux‐en‐Y gastric bypass (RYGB).

### 2.4 Data Extraction

Two authors (B.R. and A.B.) independently extracted data on the baseline characteristics of participants (e.g., age, gender, baseline BMI), and clinical outcomes, including TBWL%, resolution of MASH without worsening of fibrosis and a decrease of at least one stage of fibrosis without worsening of MASH. Conflicts in data extraction were resolved by a third investigator (M.M.).

### 2.5 Risk of Bias Assessment

The risk of bias was assessed using the Cochrane tool for assessing risk of bias in RCTs [17]. Seven domains were considered: random sequence generation, allocation concealment, blinding of participants and personnel, blinding of outcome assessment, incomplete outcome data, selective reporting and other biases. Each domain was rated as ‘low’, ‘high’, or ‘uncertain’ risk of bias. Two reviewers (A.B. and B.R.) independently assessed risk of bias, with discrepancies resolved by a third reviewer (M.M.).

### 2.6 Endpoints

*Primary Endpoint*: resolution of MASH (defined as the absence of ballooning [score of 0], no or mild inflammation [score of 0 or 1] and possible steatosis [score of 0 to 3]) with no worsening of fibrosis (defined as the absence of any increase in fibrosis stage).

*Secondary Endpoints*: improvement in fibrosis (≥ 1‐stage decrease) with no worsening of MASH (no increase in any of the NAFLD activity sub‐scores); Controlled Attenuation Parameter (CAP) at endpoint; liver stiffness at endpoint.

TBWL, expressed as % of baseline, was used as a moderator of the effects of treatment on primary and secondary endpoints.

### 2.7 Statistical Methods

For continuous variables, mean differences and 95% confidence intervals (95% CI) were calculated. For categorical outcomes, Mantel–Haenszel Odds Ratios (MH‐OR) were computed using random‐effects models. When standard errors (SEs) were available, standard deviations (SDs) were derived using the formula SD = SE × √*n*. When least‐squares means were reported, SDs were reconstructed according to methods described in the Cochrane Handbook for Systematic Reviews of Interventions (http://handbook‐5‐1.cochrane.org/chapter_7/7_7_3_2_obtaining_standard_deviations_from_standard_errors_and.htm).

Funnel plots were used for endpoints with at least 10 RCTs to assess potential publication bias.

Meta‐regression analyses were also performed for both endpoints—MASH resolution without worsening of fibrosis and improvement in fibrosis stage without worsening of MASH—to assess the potential contribution of weight loss to these outcomes.

### 2.8 NMA

NMA was conducted using a frequentist framework [18]. For each outcome, a random‐effects NMA was performed to compare all interventions (LSI, OMM, MBS and EBP) with the reference category, Pbo/SoC. These analyses enable indirect comparisons when direct trials are unavailable, by using differences from standard comparators and then combining direct and indirect comparisons to obtain a final effects estimate. League tables were used to report odds ratios (ORs) and corresponding 95% CIs for all interventions relative to Pbo/SoC for the primary outcome.

#### 2.8.1 Network Geometry and Assessment of Transitivity

The network structure was visualised using diagrams representing interventions as nodes and comparisons as links. The size of the nodes and the thickness of the edges indicated the number of studies and participants involved in each comparison. For indirect comparisons (e.g., A vs. C, B vs. C), the assumption of transitivity was assessed by comparing effect modifiers (mean age, BMI) across the studies. Network meta‐regressions (NMR) were used to explore the influence of these effect modifiers, whenever possible.

#### 2.8.2 Heterogeneity and Consistency

The τ^2^ statistic was calculated for each NMA comparison to estimate the between‐study variance (heterogeneity) across networks. The consistency of direct and indirect evidence was evaluated for the primary outcome to verify that discrepancies between estimates were minimal [19].

### 2.9 Risk of Bias and Evidence Credibility

The credibility of the evidence was assessed using the Grading of Recommendations Assessment, Development and Evaluation (GRADE) system, adapted for NMA. GRADEpro GDT software was used for this assessment, and CINeMA was employed for evaluating the NMA results. Evidence quality was rated as ‘no concerns’, ‘some concerns’, or ‘major concerns’ across six domains: within‐study risk of bias, indirectness, imprecision, heterogeneity, inconsistency and reporting bias.

### 2.10 Software

Different software platforms were used for complementary purposes: pairwise meta‐analyses and data management Review Manager (RevMan), Version 5.3 (Copenhagen: The Nordic Cochrane Centre, The Cochrane Collaboration, 2014), and meta‐regression analyses (Comprehensive Meta‐Analysis V.2; Biostat Inc. US), frequentist NMA (MetaInsight v.6.0.0—https://crsu‐metainsight.le.ac.uk/MetaInsight/), and evaluation of evidence credibility GRADEpro GDT (McMaster University, 2015 [12]; available at gradepro.org) and CINeMA—https://cinema.ispm.unibe.ch/#. All quantitative syntheses were performed within a consistent frequentist analytical framework.

## 3 Results

### 3.1 Trial Retrieval

**FIGURE 1:**
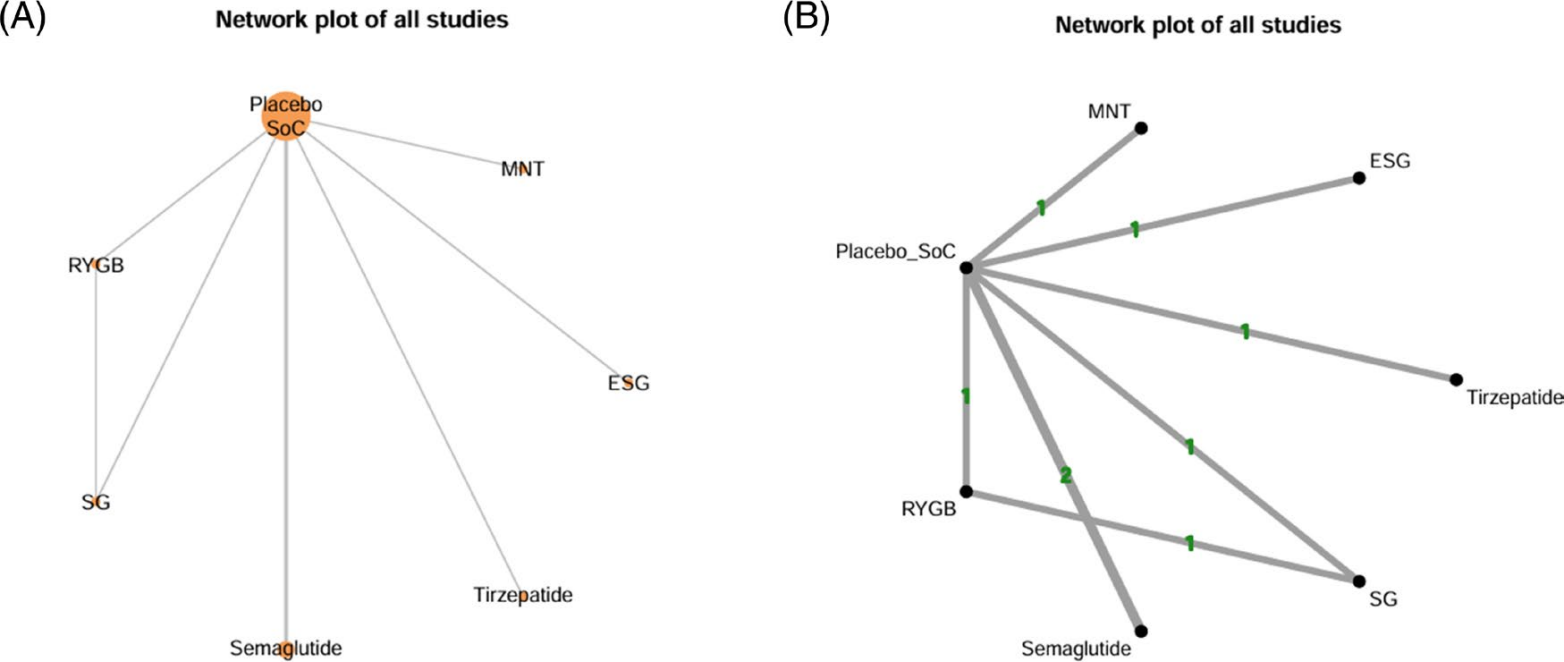
Network plot of all available studies on MASH resolution without worsening liver fibrosis (Panel A: the size of the nodes and thickness of edges represent the number of studies that examined a treatment and compared two given treatments, respectively; Panel B: numbers on the line indicate the number of trials conducted for the comparison. The shaded areas indicate that multi‐arm trials exist between the comparisons). ESG: endoscopic sleeve gastroplasty; MNT: medical nutrition therapy (i.e., structured LCD and physical exercise); Placebo/SoC: either placebo or Standard of Care.

The flow of trials included in this analysis is depicted in Figure 1S. Our search of the CENTRAL, Medline and Embase databases identified a total of 6 trials that met all inclusion criteria. These trials were performed on any approved obesity management intervention compared against either placebo/standard of care (PBO/SoC) or other active treatments. The interventions studied included one trial each for EBP, MBS and MNT, and three trials for OMM. A summary of studies excluded from the analysis, along with reasons for exclusion, is provided in Table 3S.

All trials [13, 14, 15, 20, 21, 22] enrolled patients with biopsy‐proven MASH/NASH (non‐alcoholic steatohepatitis) with blinded histologic adjudication (i.e., pathologists analysing liver biopsies were unaware of trial‐group assignments). Detailed characteristics of the included studies (including the description of histologic endpoints) are presented in Table 4S.

**FIGURE 2:**
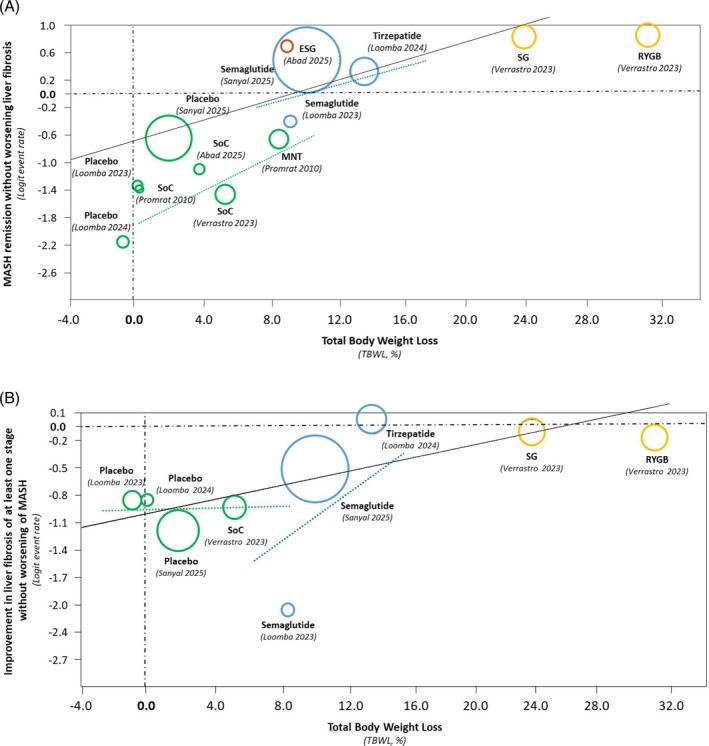
Meta‐regression analysis of randomised controlled trials assessing the relationship between the proportion of MASH resolution without worsening liver fibrosis (event rate; Panel A) and improvement of liver fibrosis without worsening MASH (event rate; Panel B), and the percentage of total body weight loss (TBWL%). Treatment categories are colour‐coded as follows: green, placebo/standard of care (SoC) and medical nutrition therapy (MNT); blue, obesity management medications (OMM); yellow, metabolic and bariatric surgery (MBS); red, endoscopic bariatric procedures (EBP). Regression lines are shown as follows: black, overall; dotted green, placebo/SoC and MNT; blue, OMM; yellow, MBS. ESG: endoscopic sleeve gastroplasty; RYGB: Rou‐en‐Y gastric bypass; SG: sleeve gastrectomy.

Study quality was found to be heterogeneous, with variability in trial design and methodological rigour. Notably, both the surgical [15] and the endoscopic trials [22] were open‐label. The remaining trials were double‐blinded, with no evidence of attrition bias or issues related to allocation or blinding (Figures 2S and 3S).

### 3.2 NMA and MA of RCTs

The evidence network was sparse and weakly connected, with five of six trials comparing active interventions only against placebo or standard care and a single head‐to‐head comparison. As a result, most treatment contrasts were based on indirect evidence, leading to wide confidence intervals and limited precision.

#### 3.2.1 Primary Endpoint: MASH Resolution Without Worsening of Fibrosis

Across the included trials, 21 pairwise comparisons were performed, involving 1321 participants. Of the six RCTs, four used MASH resolution without fibrosis worsening as the primary endpoint, while the remaining two used improvement in NAS [20] and improvement in fibrosis [21] as primary endpoints, with MASH resolution specified as a secondary outcome (Table 5S).

Traditional MAs comparing investigational treatments with placebo/SoC demonstrated a superiority of tirzepatide, semaglutide, RYGB and SG in achieving MASH resolution without worsening liver fibrosis (Figure 4S).

A network geometry plot (Figure 1) shows direct comparisons only for MBS (SG vs. RYGB) [15]. Frequentist‐NMA (Figure 5S) and the NMA league table (Table 6S) confirmed that tirzepatide, RYGB, SG and semaglutide were superior to placebo/SoC in terms of the primary endpoint, with no significant between‐group differences and no relevant inconsistencies (Table 7S).

A meta‐regression analysis was performed to examine the relationship between TBWL% and MASH resolution. The analysis revealed a significant linear correlation: each 1% increase in TBWL corresponded to a 7% higher probability of achieving MASH resolution (slope = 0.07, 95% CI, 0.05–0.09; *p* < 0.001) (Figure 2, Panel A).

### 3.3 Secondary Endpoints

Only some of the trials reported data on the improvement of liver fibrosis without worsening of MASH [13, 14, 15, 21], CAP [14, 22] or liver stiffness [13, 14, 21, 22] suggesting beneficial effects of all classes of treatment (Figures 6S and 7S). The small number of available studies was insufficient for a reliable NMA.

A meta‐regression analysis showed a significant positive association between TBWL% and improvement in liver fibrosis (Figure 2, Panel B). Specifically, each 1% increase in TBWL corresponded to a 4% higher probability of both MASH resolution and liver fibrosis improvement (slope = 0.04, 95% CI, 0.02–0.05; *p* < 0.001).

### 3.4 Risk of Bias and Confidence of Evidence

The certainty of the evidence evaluated by CINeMA for the primary endpoint for all comparisons is presented in Table 8S. The confidence of evidence was moderate for all comparisons between OMMs and the reference category, and low for EBP and MBS.

## 4 Discussion

In this systematic review and NMA of randomised clinical trials evaluating approved anti‐obesity interventions in individuals with biopsy‐confirmed MASH, we found that treatments designed for weight loss significantly increased the likelihood of achieving MASH resolution without worsening fibrosis.

NMA are typically performed to compare multiple treatments, even when head‐to‐head trials are not available for many comparisons. In the present case, NMA suggests that treatments, semaglutide and surgical interventions could be more effective than the other weight‐reducing therapies with respect to MASH resolution without worsening of fibrosis. However, such results should be considered very cautiously because of the small number of available trials and of the limited number of available direct comparisons (in fact, all trials compare the active treatment with placebo or SoC, except one [15]).

Across interventions, the magnitude of TBWL emerged as a strong predictor of histological improvement for both the liver outcomes assessed (i.e., resolution of MASH without worsening of fibrosis, and improvement of fibrosis without worsening of MASH), supporting the central mechanistic role of weight reduction in modifying the trajectory of MASH. Each 1% increment in TBWL corresponded to a 7% higher probability of MASH resolution and a 4% higher probability of fibrosis improvement. The beneficial effect of weight loss per se on MASH is in line with epidemiological studies, which show that subjects with obesity who lose more than 7%–10% of their body weight experience a more favourable evolution of the disease [10, 11]. These data align with mechanistic studies showing that reductions in visceral adiposity, improvements in insulin sensitivity and attenuation of systemic inflammation are major drivers of hepatic histological improvement [11, 23, 24]. The apparent plateau above around 20% TBWL is driven largely by two surgical arms of a single trial [15] and may reflect limited data, rather than a true biological breakpoint.

Interestingly, the linear relationship between percent weight loss and liver outcomes is evident only up to a weight loss of about 20%, both for MASH and fibrosis. However, only two treatment arms, both surgical, exceeded that threshold of weight loss; the result could therefore have been determined by chance.

Experimental data suggest that some treatments for obesity could have specific direct effects on MASH, beyond those determined by weight loss alone [25], although the expression of GLP‐1 and GIP receptors on hepatocytes or other hepatic cell types has been questioned [26]. The results of both NMA and meta‐regression do not support the hypothesis of a specific action of either GLP‐1 RA or GLP‐1/GIP dual agonists on MASH, apart from the effects of weight loss. However, these results should be interpreted very cautiously, because of the limited number of parent studies and of enrolled patients. In addition, one of the studies on semaglutide [21], with an apparently divergent result on fibrosis, enrolled a population including subjects with stage 4 fibrosis, who are less likely to benefit from any treatment. This single trial could therefore introduce a distortion, underestimating the effect of semaglutide in comparison with other treatments. On the other hand, it is also possible that expectations on direct therapeutic effects on the liver of drugs developed for obesity are overestimated. Among drugs currently under development, dual GLP‐1/glucagon agonists are proposed as treatment for both obesity and MAFLD/MASH, considering the increase in hepatic fat utilisation induced by the stimulation of glucagon receptors [27]. However, in the first trial providing data on MASH resolution and fibrosis [28], survodutide did not appear to produce benefits greater than those expected on the basis of the observed weight loss.

Several important limitations must be emphasised. First, the network was weakly connected and predominantly placebo‐anchored, with only one direct head‐to‐head comparison, substantially limiting the interpretability of indirect estimates and treatment rankings. Second, substantial clinical and methodological heterogeneity was present across trials, including differences in fibrosis stage, population characteristics and primary endpoints. Third, the meta‐regression analyses were conducted at the study‐arm level and are therefore inherently ecological. Consequently, the observed associations between TBWL% and histologic outcomes are highly susceptible to ecological bias and were strongly influenced by a small number of surgical arms achieving very large weight losses. It cannot be determined whether individuals who lost more weight were the same individuals who experienced histologic improvement. As a result, the reported slope of 7% increase in MASH resolution per 1% TBWL is likely overinterpreted and may be distorted by the few high‐TBWL arms included in the analysis. For these reasons, the meta‐regression findings should be considered exploratory and hypothesis‐generating only, and not indicative of causal effects.

A further major limitation is represented by the heterogeneity in study protocols, endpoint definition (with two studies reporting MASH resolution as a secondary endpoint [20, 21]), and the small number of eligible RCTs, enrolling relatively few subjects. Finally, the network comprised only a single head‐to‐head comparison (SG vs. RYGB) [15]; therefore, inconsistency testing, as well as treatment rankings and transitivity assessments, was inherently limited and warrants cautious interpretation.

The small number and size of available studies are a consequence of the deliberate choice, specified a priori in the PROSPERO‐registered protocol, to restrict the analysis to trials performed on biopsy‐confirmed MASH, providing results on biopsy‐confirmed endpoints (most notably, resolution of MASH without worsening of fibrosis); in addition, all the included studies employed blinded adjudication, where pathologists analysing liver biopsies were unaware of trial‐group assignments and the clinical characteristics of the participants. Broadening eligibility criteria post hoc to include non‐approved agents or non‐histologic outcomes would have represented a substantive deviation from the registered protocol and could have introduced selective inclusion and outcome‐switching bias. Therefore, we consider the rigour of diagnostic methods and criteria a major strength of this analysis. Other strengths of this work include the use of frequentist network meta‐analytic methods to enable cross‐modality comparisons and the incorporation of meta‐regression to quantify the contribution of weight loss to liver outcomes. The pre‐registered protocol and GRADE‐based assessment further enhance transparency and credibility.

In conclusion, across six biopsy‐confirmed randomised trials, interventions producing greater weight loss were associated with higher rates of MASH resolution and fibrosis improvement. However, these observations arise from a sparse, heterogeneous and largely indirect evidence network, and from exploratory, study‐level analyses that preclude causal inference. Head‐to‐head, adequately powered randomised trials remain essential to define modality‐specific and potential weight‐independent effects.

Overall, the findings highlight weight reduction as a central mechanistic target in MASH management; however, they do not establish the relative efficacy of distinct obesity‐management modalities, despite their prioritisation in several clinical algorithms and guidelines [29, 30, 31, 32, 33]. Definitive assessment of weight‐independent effects and modality‐specific benefits will require adequately powered, head‐to‐head RCTs across diverse patient populations, including those with advanced fibrosis. Such evidence will be essential to support precision treatment selection and to optimise outcomes in this rapidly evolving therapeutic landscape.

## Author Contributions

M.M. was responsible for the conception and design of the study, data acquisition, data analysis and drafting and final approval of the manuscript. A.B., S.B., L.B. and R.C. contributed to data acquisition and to drafting and final approval of the manuscript. M.D.L., A.G., E.M., T.Z.P., P.S. and D.D. contributed to drafting and final approval of the manuscript.

## Funding

The authors have nothing to report.

## Conflicts of Interest

Matteo Monami and Edoardo Mannucci have received speaking fees from Lilly and Novo Nordisk. Luca Busetto has received payment of honoraria from Eli Lilly, Novo Nordisk, Boehringer Ingelheim, Pfizer, Bruno Farmaceutici, Regeneron, Rythm Pharmaceuticals and Pronokal as speaker and/or member of advisory boards. Dror Dicker has received speaker and advisory board fees from Boehringer‐Ingelheim, Eli‐Lilly, Novo Nordisk, Astra Zeneca and research grants from Eli Lilly, NovoNordisk and Boehringer Ingelheim. Paolo Sbraccia received payment of honoraria and consulting fees from Boehringer Ingelheim, Chiesi, Novo Nordisk, Eli Lilly, Pfizer and Roche as a member of advisory boards.

## Supporting information


**Figure 1S.** Trial flow summary.
**Figure 2S**. Risk of bias graph: review authors' judgements about each risk of bias item presented as percentages across all included studies.
**Figure 3S**. Risk of bias summary: review authors' judgements about each risk of bias item for each included study.
**Figure 4S**. Individual study results grouped by treatment comparison for MASH remission without worsening liver fibrosis.
**Figure 5S**. Frequentist‐network meta‐analysis for all available treatments using placebo/conventional diet as the reference category on MASH remission without worsening liver fibrosis.
**Figure 6S**. Individual study results for improvement of liver fibrosis of at least one stage without worsening MASH.
**Figure 7S**. Individual study results grouped by treatment categories for endpoint liver stiffness (kPa; Panel A) and Controlled Attenuation Parameter (CAP; Panel B).
**Figure 8S**. Overall NMA risk of bias (within‐study and reporting bias, indirectness, imprecision, heterogeneity, and incoherence) for each comparison, versus the reference category (i.e., LSI/Placebo/None).
**Table 1S**. PRISMA extension for Network Meta‐Analysis checklist.
**Table 2S**. Detailed information on search strategy.
**Table 3S**. Excluded trials and reasons for the exclusion.
**Table 4S**. Principal baseline characteristics of the included studies.
**Table 5S**. Principal characteristics of the network of all available studies on MASH remission.
**Table 6S**. NMA league table.
**Table 7S**. Assessment of inconsistency across all studies.
**Table 8S**. Evaluation of confidence in the network meta‐analysis results.

## Data Availability

Data sharing not applicable to this article as no datasets were generated or analysed during the current study.
