# Supplementary material for: Weight Loss as a Determinant of Histological Improvement in Metabolic Dysfunction‐Associated Steatotic Liver Disease in People With Obesity. A Systematic Review and Network Meta‐Analysis of Randomised Clinical Trials
Source: Diabetes Obes Metab. 2026 Mar 9;28(5):4253–60. doi: 10.1111/dom.70617 (PMC13071265; doi:10.1111/dom.70617)
Supplement: Supplementary file 1 — Figure 1S. Trial flow summary. Figure 2S. Risk of bias graph: review authors' judgements about each risk of bias item presented as percentages across all included studies. Figure 3S. Risk of bias summary: review authors' judgements about each risk of bias item for each included study. Figure 4S. Individual study results grouped by treatment comparison for MASH remission without worsening liver fibrosis. Figure 5S. Frequentist‐network meta‐analysis for all available treatments using placebo/conventional diet as the reference category on MASH remission without worsening liver fibrosis. Figure 6S. Individual study results for improvement of liver fibrosis of at least one stage without worsening MASH. Figure 7S. Individual study results grouped by treatment categories for endpoint liver stiffness (kPa; Panel A) and Controlled Attenuation Parameter (CAP; Panel B). Figure 8S. Overall NMA risk of bias (within‐study and reporting bias, indirectness, imprecision, heterogeneity, and incoherence) for each comparison, versus the reference category (i.e., LSI/Placebo/None). Table 1S. PRISMA extension for Network Meta‐Analysis checklist. Table 2S. Detailed information on search strategy. Table 3S. Excluded trials and reasons for the exclusion. Table 4S. Principal baseline characteristics of the included studies. Table 5S. Principal characteristics of the network of all available studies on MASH remission. Table 6S. NMA league table. Table 7S. Assessment of inconsistency across all studies. Table 8S. Evaluation of confidence in the network meta‐analysis results. [file DOM-28-4253-s001.docx]

**Supplementary appendix**

**Supplement to:**

**Weight Loss as a Determinant of Histological Improvement in Metabolic Dysfunction-Associated Steatotic Liver Disease in people with obesity. A Systematic Review and Network Meta-analysis of Randomized Clinical Trials.**

Matteo Monami^1*^, Amanda Belluzzi^2*^, Silvio Buscemi^3^, Luca Busetto^4^, Ricardo Cohen^5^, Maurizio De Luca^2^, Andrea Galli^1^, Edoardo Mannucci^1^, Tarissa Z. Petry^5^, Benedetta Ragghianti^1^, Paolo Sbraccia^6^, Dror Dicker^7^.

Affiliations

*^1^Careggi Teaching Hospital and University of Florence, Florence, Italy; ^2^Rovigo Hospital, ULSS5 Polesana, Viale Tre Martirii, Rovigo, Italy; ^3^Department of Promozione della Salute, Materno-Infantile, Medicina Interna e Specialistica di Eccellenza (PROMISE), University of Palermo, Palermo, Italy; ^4^Department of Medicine, University of Padova, Italy; ^5^The Center for Obesity and Diabetes, Hospital Alemao Oswaldo Cruz, Sao Paulo, Brazil.; ^6^Department of Systems Medicine, University of Rome Tor Vergata, Rome, Italy; ^7^Internal Medicine D and Obesity Clinic, Hasharon Hospital-Rabin Medical Center, Faculty of Medicine, Tel-Aviv University, Tel-Aviv, Israel.*

*Equally contributed

**Table of contents**

**Figures**

[Figure 1S – Trial flow summary 3](#_heading=h.ceu5lfsm5zqv)

[Figure 2S – Risk of bias graph 4](#_heading=h.kindbjfv21dk)

[Figure 3S – Risk of bias summary 5](#_heading=h.92yt3omn0wdk)

[Figure 4S – Individual study results grouped by treatment comparison for MASH remission 6](#_heading=h.z6h3b394rjl9)

[Figure 5S – Frequentist-network meta-analysis for all available. 7](#_heading=h.7zfpokvlsk1q)

[Figure 6S – Individual study results for improvement of liver fibrosis without worsening MASH. 8](#_heading=h.vac75nosexa3)

[Figure 7S – Individual study results grouped by treatment categories for liver endpoints. 9](#_heading=h.ibm3bpf0trwp)

[**Tables** 11](#_heading=h.qjjvbxvkqvvk)

[Table 1S – PRISMA extension for Network Meta-Analysis checklist 11](#_heading=h.1mz6lyi4u0gs)

[Table 2S – Detailed information on search strategy 16](#_heading=h.wlivwuyccrdc)

[Table 3S – Excluded trials and reasons for the exclusion. 19](#_heading=h.th099qgh461h)

[Table 4S – Principal baseline characteristics of the included studies. 21](#_heading=h.hbq6938xf6yw)

[Table 5S – Principal characteristics of the network of all available studies on MASH remission 22](#_heading=h.ouiihzku6hcr)

[Table 6S – NMA league table 23](#_heading=h.qi0c3kdzfzq4)

[Table 7S – Assessment of inconsistency across all studies. 24](#_heading=h.1bypjllt9c2z)

Table 8S – Evaluation of confidence in the network meta-analysis results [25](#_heading=h.1bypjllt9c2z)

[**References** 26](#_heading=h.of08ukitveov)

# Figure 1S – Trial flow summary


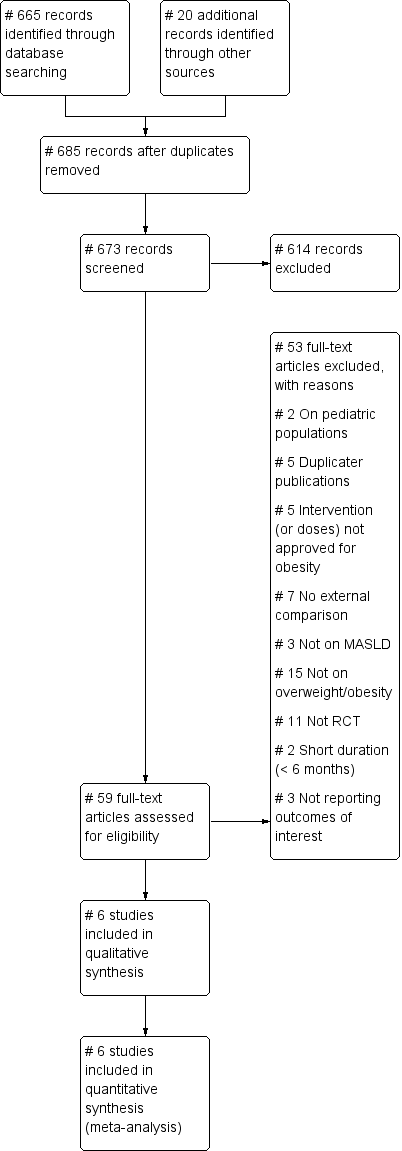


# Figure 2S – Risk of bias graph: review authors' judgements about each risk of bias item presented as percentages across all included studies.

**
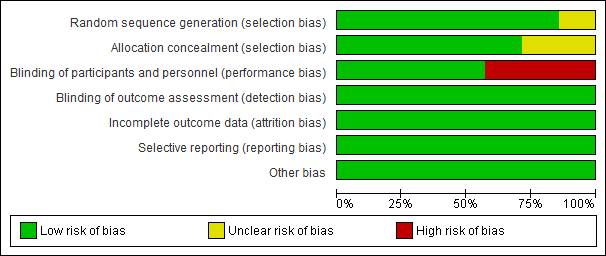
**

# Figure 3S – Risk of bias summary: review authors' judgements about each risk of bias item for each included study.

**
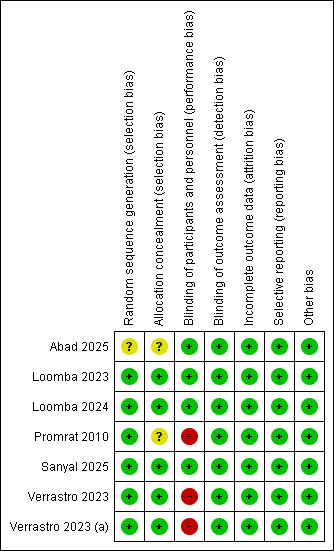
**

# Figure 4S – Individual study results grouped by treatment comparison for MASH remission without worsening liver fibrosis.


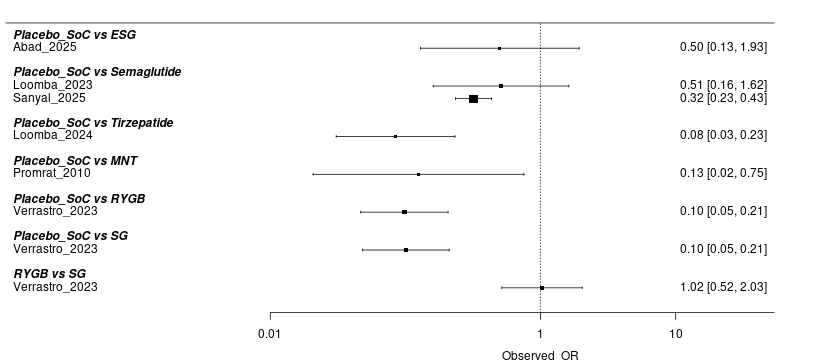


# Figure 5S – Frequentist-network meta-analysis for all available treatments using placebo/conventional diet as the reference category on MASH remission without worsening liver fibrosis.


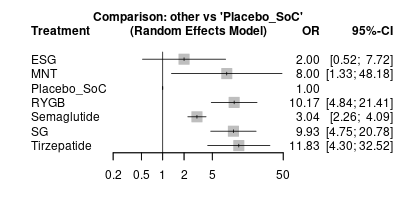


# Figure 6S – Individual study results for improvement of liver fibrosis of at least one stage without worsening MASH.


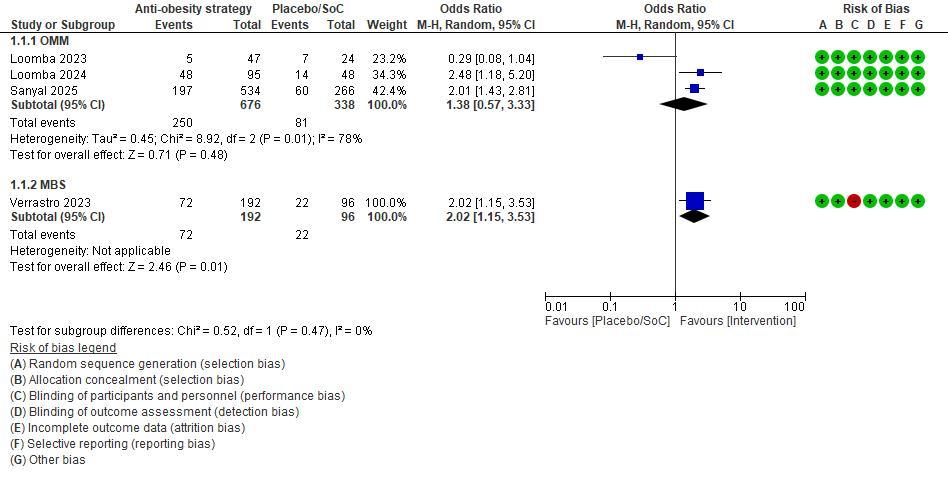


# Figure 7S – Individual study results grouped by treatment categories for endpoint liver stiffness (kPa; Panel A) and Controlled Attenuation Parameter (CAP; Panel B).

**A**


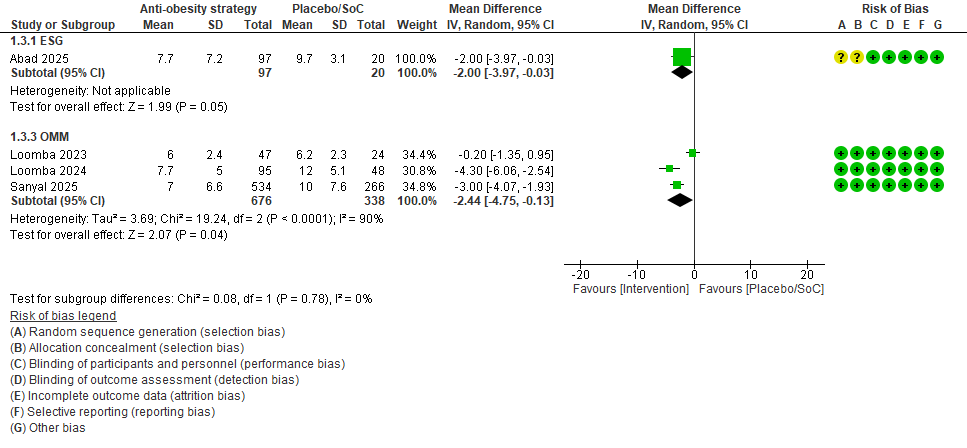


**B**

**
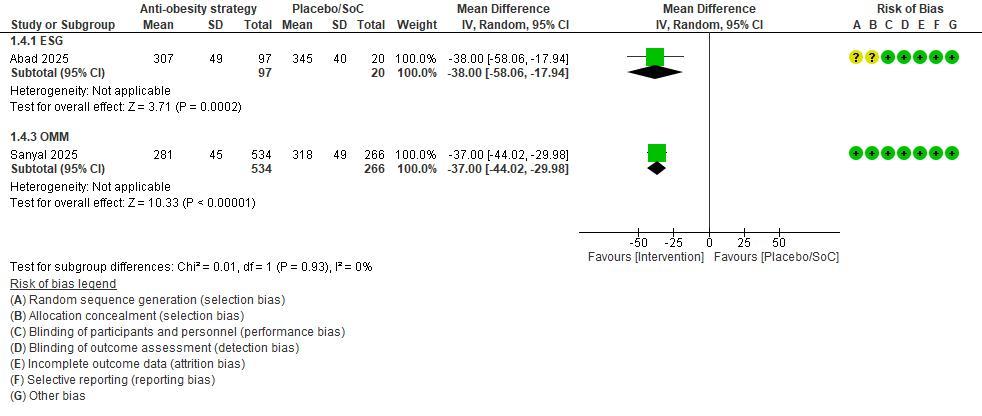
**

Figure 8S – Overall NMA risk of bias (within-study and reporting bias, indirectness, imprecision, heterogeneity, and incoherence) for each comparison, versus the reference category (i.e., LSI/Placebo/None).

**A**


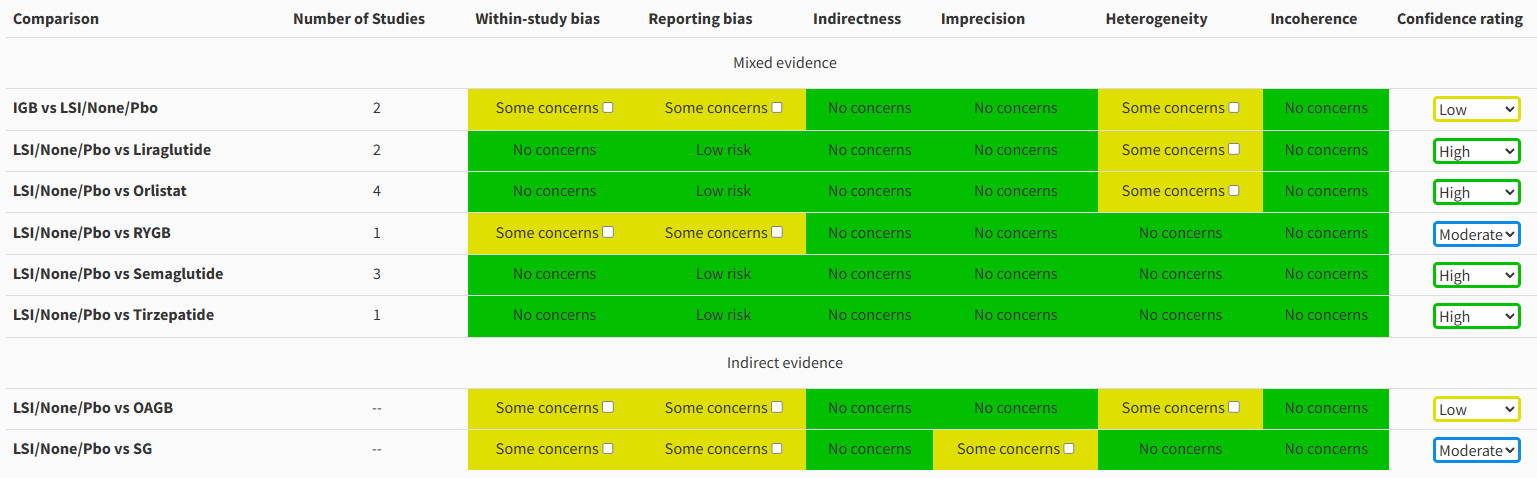


# TABLES

# Table 1S – PRISMA extension for Network Meta-Analysis checklist.

| **Section/topic** | **#** | **Checklist item** | **Reported on page #** |
| --- | --- | --- | --- |
| Title | 1 | Effects of anti-obesity strategies (lifestyle, pharmacological, and surgical interventions) on Metabolic Dysfunction-Associated Steatotic Liver Disease in people with obesity: A meta-analysis and network meta-analysis of randomized controlled studies. | Title page |
| Abstract | 2 | Background: Metabolic dysfunction–associated steatotic liver disease (MASLD) is closely linked to obesity and insulin resistance, and sustained weight loss is associated with histological improvement. Whether different obesity-management modalities exert weight-independent hepatic effects remains uncertain.  Methods: We conducted a systematic review and network meta-analysis (NMA) of randomized controlled trials evaluating lifestyle intervention, obesity management medications, endoscopic sleeve gastroplasty, and metabolic and bariatric surgery in adults with BMI ≥27 kg/m² and biopsy-confirmed MASH. The primary endpoint was MASH resolution without worsening of fibrosis. Study-level meta-regressions explored associations between total body weight loss (TBWL%) and histologic outcomes.  Results: Six RCTs (n = 1,379) met inclusion criteria. Tirzepatide, semaglutide, sleeve gastrectomy, and Roux-en-Y gastric bypass were superior to placebo or standard care for achieving MASH resolution. Because the network was weakly connected and largely placebo-anchored, indirect estimates were imprecise. Across study arms, greater TBWL% was associated with higher rates of MASH resolution and fibrosis improvement; however, these associations were strongly influenced by a small number of high-weight-loss surgical arms.  Conclusions: Weight loss was consistently associated with histologic improvement across available RCTs. However, the limited evidence base, sparse network structure, and ecological nature of the meta-regression preclude causal inference. These findings should be considered exploratory and hypothesis-generating, underscoring the need for adequately powered head-to-head trials. | Abstract |
| Rationale | 3 | This study presents the results of a systematic review (SR) followed by a network meta-analysis (NMA) of randomized clinical trials (RCTs) that compare lifestyle interventions (LSI), obesity management medications (OMM), endoscopic bariatric procedures (EBS), and metabolic bariatric surgery (MBS) against placebo/standard of care or other active comparators in individuals with overweight or obesity. | Introduction |
| Objectives | 4 | Specifically, this NMA aims to: 1) provide healthcare professionals with a comprehensive assessment of the efficacy and safety of available approved treatments in relation to liver outcomes, and 2) evaluate the relationship between weight loss and improvements in liver outcomes, using the highest quality evidence as required by GRADE methodology. | Introduction |
| Protocol and registration | 5 | The study has been uploaded on the PROSPERO website (#CRD420251148787). | Methods |
| Eligibility criteria | 6 | Randomized controlled trials (RCTs) that enrolled patients with a body mass index (BMI) greater than 27 kg/m² and steatohepatitis were included. These trials compared LSI (based on Medical Nutrition Therapy [MNT], including any low-calorie diets [LCD] or very low-calorie diets [VLCD] and structured physical activity programs), OMM, EBP, and MBS against placebo/standard care (Pbo/SoC) or compared two active treatments. Eligible trials were required to have a minimum follow-up of 26 weeks. No language or date restrictions were applied. | Methods |
| Information sources | 7 | A comprehensive literature search was conducted in Medline, Embase, and the Cochrane Central Register of Controlled Trials (CENTRAL) up until November 1, 2025. | Methods |
| Search | 8 | See Table 2S. | Table 2S |
| Study selection | 9 | Paired reviewers (B.R. and A.B.) independently screened titles, abstracts, and full-text manuscripts, extracting relevant data from studies meeting the inclusion and exclusion criteria. | Methods |
| Data collection process | 10 | The principal characteristics of the included trials included were reported in Suppl Mat. | Table 3S |
| Data items | 11 | Two authors (B.R., A.B.) independently extracted data on the baseline characteristics of participants (e.g., age, gender, baseline BMI), and clinical outcomes, including total body weight loss (TBWL%), resolution of metabolic dysfunction-associated steatohepatitis (MASH) without worsening of fibrosis, and decrease of at least one stage of fibrosis without worsening of MASH. | Methods |
| Risk of bias in individual studies | 12 | The risk of bias was assessed using the Cochrane tool for assessing risk of bias in RCTs [14]. Seven domains were considered: random sequence generation, allocation concealment, blinding of participants and personnel, blinding of outcome assessment, incomplete outcome data, selective reporting, and other biases. Each domain was rated as "low," "high," or "uncertain" risk of bias. Two reviewers (A.B. and B.R.) independently assessed risk of bias, with discrepancies resolved by a third reviewer (M.M.). | Methods |
| Summary measures | 13 | For continuous variables, mean differences and 95% confidence intervals (95% CI) were calculated. For categorical outcomes, Mantel–Haenszel Odds Ratios (MH-OR) were computed using random-effects models. | Methods |
| Planned methods of analysis | 14 | NMA was conducted using a frequentist framework. For each outcome, a random-effects NMA was performed to compare all interventions (LSI, OMM, MBS, and EBP) with the reference category, Pbo/SoC. | Methods – Statistical analysis |
| Network geometry | S1 | The network structure was visualized using diagrams representing interventions as nodes and comparisons as links. The size of the nodes and the thickness of the edges indicated the number of studies and participants involved in each comparison. | Methods – Network geometry |
| Assessment of transitivity | S2 | For indirect comparisons (e.g., A vs. C, B vs. C), the assumption of transitivity was assessed by comparing effect modifiers (mean age, BMI) across the studies. Network meta-regressions (NMR) were used to explore the influence of these effect modifiers, whenever possible. | Methods – Network geometry |
| Risk of bias across studies | 15 | The credibility of the evidence was assessed using the Grading of Recommendations Assessment, Development, and Evaluation (GRADE) system, adapted for NMA. GRADEpro GDT software was used for this assessment, and CINeMA was employed for evaluating the NMA results. | Methods |
| Additional analyses | 16 | Meta-regression analyses were also performed for both endpoints—MASH resolution without worsening of fibrosis and improvement in fibrosis stage without worsening of MASH— to assess the potential contribution of weight loss to these outcomes. | Methods |
| Study selection | 17 | The search of Medline and Embase database allowed the identification of 6 trials (i.e., 21 pairwise comparisons). | Results / Figure 1S |
| Study characteristics | 18 | The principal characteristics of included trials were reported in Supplementary Materials. | Table 4S |
| Risk of bias within studies | 19 | The quality of studies was heterogeneous. | Figures 2S–3S |
| Results of individual studies | 20 | Traditional MAs comparing investigational treatments with placebo/SoC demonstrated a superiority of tirzepatide, semaglutide, RYGB, and SG in achieving MASH remission without worsening liver fibrosis and improvement of liver fibrosis without worsening of MASH. | Figures 4S–7S |
| Synthesis of results | 21 | Frequentist-NMA and the NMA league table confirmed that tirzepatide, RYGB, SG, and semaglutide were superior to placebo/SoC in terms of the primary endpoint, with no significant between-group differences and no relevant inconsistencies. | Results / Table 6S |
| Inconsistency | S3 | Describe methods used to assess inconsistency. | Methods / Table 7S |
| Risk of bias across studies | 22 | The certainty of the evidence evaluated by CINeMA for the primary endpoint for all comparisons is presented in Figure of the Supplementary Materials. The confidence of evidence was moderate for all comparisons between OMMs and the reference category, and low for EBP and MBS. | Results / Table 8S |
| Additional analysis | 23 | A meta-regression analysis was performed to examine the relationship between total body weight loss (TBWL%) and MASH remission. The analysis revealed a significant linear correlation: each 1% increase in TBWL corresponded to a 7% higher probability of achieving MASH remission (slope = 0.07, 95% CI, 0.05–0.09; p < 0.001). A meta-regression analysis showed a significant positive association between TBWL% and improvement in liver fibrosis. Specifically, each 1% increase in TBWL corresponded to a 4% higher probability of both MASH remission and liver fibrosis improvement (slope = 0.04, 95% CI, 0.02–0.05; p < 0.001). | Results – Meta-regression |
| Summary of evidence | 24 | In this systematic review and network meta-analysis of randomized clinical trials evaluating approved anti-obesity interventions in individuals with biopsy-confirmed MASH, we found that treatments designed for weight loss significantly increased the likelihood of achieving MASH resolution without worsening fibrosis. | Discussion |
| Limitations | 25 | Limitations were reported in extenso in the Discussion section. | Discussion |
| Conclusions | 26 | In conclusion, across six biopsy‑confirmed randomized trials, interventions producing greater weight loss were associated with higher rates of MASH resolution and fibrosis improvement. However, these observations arise from a sparse, heterogeneous, and largely indirect evidence network, and from exploratory, study‑level analyses that preclude causal inference. Head‑to‑head, adequately powered randomized trials remain essential to define modality‑specific and potential weight‑independent effects. | Discussion |
| Funding | 27 | This research was performed as a part of the institutional activity of the unit, with no specific funding. | Funding |

# Table 2S – Detailed information on search strategy

| **Limits:** Human studies; any date up to November 1^st^, 2025 |
| --- |
| Search string: (orlistat OR naltrexone OR bupropion OR liraglutide OR semaglutide OR tirzepatide OR MBS OR Sleeve gastrectomy OR Roux-en-Y gastric bypass OR one anastomosis gastric bypass OR laparoscopic adjustable gastric banding OR biliopancreatic diversion OR single anastomosis duodenal-ileal bypass OR greater curvature plication OR Intragastric balloons OR Primary Obesity Surgery Endoluminal OR endoscopic sleeve gastroplasty) AND obesity AND (MASLD OR MASH OR NASH OR stetaohepatitis OR hepatitis)  ***Pubmed***  ("orlistat"[Supplementary Concept] OR "orlistat"[All Fields] OR "orlistat"[MeSH Terms] OR "orlistat s"[All Fields] OR ("naltrexone"[Supplementary Concept] OR "naltrexone"[All Fields] OR "naltrexon"[All Fields] OR "naltrexone"[MeSH Terms] OR "naltrexone s"[All Fields]) OR ("bupropion"[Supplementary Concept] OR "bupropion"[All Fields] OR "amfebutamone"[All Fields] OR "bupropion"[MeSH Terms] OR "bupropion s"[All Fields] OR "bupropione"[All Fields]) OR ("liraglutid"[All Fields] OR "liraglutide"[Supplementary Concept] OR "liraglutide"[All Fields] OR "liraglutide"[MeSH Terms] OR "liraglutide s"[All Fields]) OR ("semaglutide"[Supplementary Concept] OR "semaglutide"[All Fields]) OR ("tirzepatide"[Supplementary Concept] OR "tirzepatide"[All Fields] OR "tirzepatide"[MeSH Terms]) OR "MBS"[All Fields] OR (("sleeve"[All Fields] OR "sleeved"[All Fields] OR "sleeves"[All Fields] OR "sleeving"[All Fields]) AND ("gastrectomy"[MeSH Terms] OR "gastrectomy"[All Fields] OR "gastrectomies"[All Fields])) OR ("gastric bypass"[MeSH Terms] OR ("gastric"[All Fields] AND "bypass"[All Fields]) OR "gastric bypass"[All Fields] OR "roux en y gastric bypass"[All Fields]) OR ("one"[All Fields] AND ("anastomosis surgical"[MeSH Terms] OR ("anastomosis"[All Fields] AND "surgical"[All Fields]) OR "surgical anastomosis"[All Fields] OR "anastomosis"[All Fields]) AND ("gastric bypass"[MeSH Terms] OR ("gastric"[All Fields] AND "bypass"[All Fields]) OR "gastric bypass"[All Fields])) OR (("laparoscopes"[MeSH Terms] OR "laparoscopes"[All Fields] OR "laparoscope"[All Fields] OR "laparoscopical"[All Fields] OR "laparoscopically"[All Fields] OR "laparoscopics"[All Fields] OR "laparoscopy"[MeSH Terms] OR "laparoscopy"[All Fields] OR "laparoscopic"[All Fields]) AND ("adjustability"[All Fields] OR "adjustable"[All Fields] OR "adjustables"[All Fields] OR "adjustible"[All Fields]) AND ("gastrics"[All Fields] OR "stomach"[MeSH Terms] OR "stomach"[All Fields] OR "gastric"[All Fields]) AND ("banded"[All Fields] OR "banding"[All Fields] OR "bandings"[All Fields])) OR ("biliopancreatic diversion"[MeSH Terms] OR ("biliopancreatic"[All Fields] AND "diversion"[All Fields]) OR "biliopancreatic diversion"[All Fields]) OR (("single person"[MeSH Terms] OR ("single"[All Fields] AND "person"[All Fields]) OR "single person"[All Fields] OR "single"[All Fields] OR "singles"[All Fields]) AND ("anastomosis surgical"[MeSH Terms] OR ("anastomosis"[All Fields] AND "surgical"[All Fields]) OR "surgical anastomosis"[All Fields] OR "anastomosis"[All Fields]) AND "duodenal-ileal"[All Fields] AND ("bypass"[All Fields] OR "bypassed"[All Fields] OR "bypasses"[All Fields] OR "bypassing"[All Fields])) OR ("greater"[All Fields] AND ("curvature"[All Fields] OR "curvatures"[All Fields]) AND ("plicate"[All Fields] OR "plicated"[All Fields] OR "plicates"[All Fields] OR "plicating"[All Fields] OR "plication"[All Fields] OR "plications"[All Fields] OR "plicator"[All Fields])) OR (("intragastral"[All Fields] OR "intragastrally"[All Fields] OR "intragastric"[All Fields] OR "intragastrical"[All Fields] OR "intragastrically"[All Fields]) AND ("balloon"[All Fields] OR "balloon s"[All Fields] OR "balloons"[All Fields])) OR (("primaries"[All Fields] OR "primary"[All Fields]) AND ("obes surg"[Journal] OR ("obesity"[All Fields] AND "surgery"[All Fields]) OR "obesity surgery"[All Fields]) AND ("endoluminal"[All Fields] OR "endoluminally"[All Fields])) OR (("endoscope s"[All Fields] OR "endoscoped"[All Fields] OR "endoscopes"[MeSH Terms] OR "endoscopes"[All Fields] OR "endoscope"[All Fields] OR "endoscopical"[All Fields] OR "endoscopically"[All Fields] OR "endoscopy"[MeSH Terms] OR "endoscopy"[All Fields] OR "endoscopic"[All Fields]) AND ("sleeve"[All Fields] OR "sleeved"[All Fields] OR "sleeves"[All Fields] OR "sleeving"[All Fields]) AND ("gastroplasty"[MeSH Terms] OR "gastroplasty"[All Fields] OR "gastroplasties"[All Fields]))) AND ("obeses"[All Fields] OR "obesity"[MeSH Terms] OR "obesity"[All Fields] OR "obese"[All Fields] OR "obesities"[All Fields] OR "obesity s"[All Fields]) AND ("MASLD"[All Fields] OR "MASH"[All Fields] OR ("non alcoholic fatty liver disease"[MeSH Terms] OR ("non alcoholic"[All Fields] AND "fatty"[All Fields] AND "liver"[All Fields] AND "disease"[All Fields]) OR "non alcoholic fatty liver disease"[All Fields] OR "nash"[All Fields]) OR ("hepatitis"[MeSH Terms] OR "hepatitis"[All Fields] OR "hepatitides"[All Fields] OR "hepatitis a"[MeSH Terms] OR "hepatitis a"[All Fields]))  Translations  orlistat: "orlistat"[Supplementary Concept] OR "orlistat"[All Fields] OR "orlistat"[MeSH Terms] OR "orlistat's"[All Fields]  naltrexone: "naltrexone"[Supplementary Concept] OR "naltrexone"[All Fields] OR "naltrexon"[All Fields] OR "naltrexone"[MeSH Terms] OR "naltrexone's"[All Fields]  bupropion: "bupropion"[Supplementary Concept] OR "bupropion"[All Fields] OR "amfebutamone"[All Fields] OR "bupropion"[MeSH Terms] OR "bupropion's"[All Fields] OR "bupropione"[All Fields]  liraglutide: "liraglutid"[All Fields] OR "liraglutide"[Supplementary Concept] OR "liraglutide"[All Fields] OR "liraglutide"[MeSH Terms] OR "liraglutide's"[All Fields]  semaglutide: "semaglutide"[Supplementary Concept] OR "semaglutide"[All Fields]  tirzepatide: "tirzepatide"[Supplementary Concept] OR "tirzepatide"[All Fields] OR "tirzepatide"[MeSH Terms]  Sleeve: "sleeve"[All Fields] OR "sleeved"[All Fields] OR "sleeves"[All Fields] OR "sleeving"[All Fields]  gastrectomy: "gastrectomy"[MeSH Terms] OR "gastrectomy"[All Fields] OR "gastrectomies"[All Fields]  Roux-en-Y gastric bypass: "gastric bypass"[MeSH Terms] OR ("gastric"[All Fields] AND "bypass"[All Fields]) OR "gastric bypass"[All Fields] OR "roux en y gastric bypass"[All Fields]  anastomosis: "anastomosis, surgical"[MeSH Terms] OR ("anastomosis"[All Fields] AND "surgical"[All Fields]) OR "surgical anastomosis"[All Fields] OR "anastomosis"[All Fields]  gastric bypass: "gastric bypass"[MeSH Terms] OR ("gastric"[All Fields] AND "bypass"[All Fields]) OR "gastric bypass"[All Fields]  laparoscopic: "laparoscopes"[MeSH Terms] OR "laparoscopes"[All Fields] OR "laparoscope"[All Fields] OR "laparoscopical"[All Fields] OR "laparoscopically"[All Fields] OR "laparoscopics"[All Fields] OR "laparoscopy"[MeSH Terms] OR "laparoscopy"[All Fields] OR "laparoscopic"[All Fields]  adjustable: "adjustability"[All Fields] OR "adjustable"[All Fields] OR "adjustables"[All Fields] OR "adjustible"[All Fields]  gastric: "gastrics"[All Fields] OR "stomach"[MeSH Terms] OR "stomach"[All Fields] OR "gastric"[All Fields]  banding: "banded"[All Fields] OR "banding"[All Fields] OR "bandings"[All Fields]  biliopancreatic diversion: "biliopancreatic diversion"[MeSH Terms] OR ("biliopancreatic"[All Fields] AND "diversion"[All Fields]) OR "biliopancreatic diversion"[All Fields]  single: "single person"[MeSH Terms] OR ("single"[All Fields] AND "person"[All Fields]) OR "single person"[All Fields] OR "single"[All Fields] OR "singles"[All Fields]  anastomosis: "anastomosis, surgical"[MeSH Terms] OR ("anastomosis"[All Fields] AND "surgical"[All Fields]) OR "surgical anastomosis"[All Fields] OR "anastomosis"[All Fields]  bypass: "bypass"[All Fields] OR "bypassed"[All Fields] OR "bypasses"[All Fields] OR "bypassing"[All Fields]  curvature: "curvature"[All Fields] OR "curvatures"[All Fields]  plication: "plicate"[All Fields] OR "plicated"[All Fields] OR "plicates"[All Fields] OR "plicating"[All Fields] OR "plication"[All Fields] OR "plications"[All Fields] OR "plicator"[All Fields]  Intragastric: "intragastral"[All Fields] OR "intragastrally"[All Fields] OR "intragastric"[All Fields] OR "intragastrical"[All Fields] OR "intragastrically"[All Fields]  balloons: "balloon"[All Fields] OR "balloon's"[All Fields] OR "balloons"[All Fields]  Primary: "primaries"[All Fields] OR "primary"[All Fields]  Obesity Surgery: "Obes Surg"[Journal:__jid9106714] OR ("obesity"[All Fields] AND "surgery"[All Fields]) OR "obesity surgery"[All Fields]  Endoluminal: "endoluminal"[All Fields] OR "endoluminally"[All Fields]  endoscopic: "endoscope's"[All Fields] OR "endoscoped"[All Fields] OR "endoscopes"[MeSH Terms] OR "endoscopes"[All Fields] OR "endoscope"[All Fields] OR "endoscopical"[All Fields] OR "endoscopically"[All Fields] OR "endoscopy"[MeSH Terms] OR "endoscopy"[All Fields] OR "endoscopic"[All Fields]  sleeve: "sleeve"[All Fields] OR "sleeved"[All Fields] OR "sleeves"[All Fields] OR "sleeving"[All Fields]  gastroplasty: "gastroplasty"[MeSH Terms] OR "gastroplasty"[All Fields] OR "gastroplasties"[All Fields]  OBESITY: "obeses"[All Fields] OR "obesity"[MeSH Terms] OR "obesity"[All Fields] OR "obese"[All Fields] OR "obesities"[All Fields] OR "obesity's"[All Fields]  NASH: "non-alcoholic fatty liver disease"[MeSH Terms] OR ("non-alcoholic"[All Fields] AND "fatty"[All Fields] AND "liver"[All Fields] AND "disease"[All Fields]) OR "non-alcoholic fatty liver disease"[All Fields] OR "nash"[All Fields]  hepatitis: "hepatitis"[MeSH Terms] OR "hepatitis"[All Fields] OR "hepatitides"[All Fields] OR "hepatitis a"[MeSH Terms] OR "hepatitis a"[All Fields]  EMBASE  (orlistat OR naltrexone OR bupropion OR liraglutide OR semaglutide OR tirzepatide OR MBS OR Sleeve gastrectomy OR Roux-en-Y gastric bypass OR one anastomosis gastric bypass OR laparoscopic adjustable gastric banding OR biliopancreatic diversion OR single anastomosis duodenal-ileal bypass OR greater curvature plication OR Intragastric balloons OR Primary Obesity Surgery Endoluminal OR endoscopic sleeve gastroplasty) AND obesity AND (MASLD OR MASH OR NASH OR stetaohepatitis OR hepatitis)  Cochrane Central Register of Controlled Trials  (orlistat OR naltrexone OR bupropion OR liraglutide OR semaglutide OR tirzepatide OR MBS OR Sleeve gastrectomy OR Roux-en-Y gastric bypass OR one anastomosis gastric bypass OR laparoscopic adjustable gastric banding OR biliopancreatic diversion OR single anastomosis duodenal-ileal bypass OR greater curvature plication OR Intragastric balloons OR Primary Obesity Surgery Endoluminal OR endoscopic sleeve gastroplasty) AND obesity AND (MASLD OR MASH OR NASH OR stetaohepatitis OR hepatitis) |
| **Additional search:**  Additional manual search of the references of included trials and former meta-analyses was carried out to identify other newly published and unpublished studies. Completed but yet unpublished studies with the procedures specified above were searched in the www.clinicaltrials.gov register. using the same search string as above. |

# Table 3S – Excluded trials and reasons for the exclusion.

| **Study**  (First autor) | **Publication**  **year** | **Reason for the exclusion** |
| --- | --- | --- |
| Abenavoli ^1^ | 2107 | Not on overweight/obesity |
| Abenavoli ^2^ | 2015 | Not on overweight/obesity |
| Alkhouri ^3^ | 2022 | No external comparison |
| Aller ^4^ | 2014 | Not on overweight/obesity |
| Argo ^5^ | 2015 | Not on overweight/obesity |
| Argo ^6^ | 2018 | Not RCT |
| Armstrong ^7^ | 2025 | Duplicate |
| Athyros ^8^ | 2006 | Not on overweight/obesity |
| Barsalani ^9^ | 2013 | Not on MASLD |
| Bomhof ^10^ | 2019 | Not on overweight/obesity |
| Chen ^11^ | 2019 | Not on overweight/obesity |
| Craven ^12^ | 2020 | Not on overweight/obesity |
| Crommen ^13^ | 2024 | Not RCT |
| De Barros ^14^ | 2020 | Short duration |
| Deibert ^15^ | 2019 | No external comparison |
| Deshmukh^16^ | 2024 | Age< 18 years |
| Dichtel ^17^ | 2021 | Not RCT |
| Dong ^18^ | 2016 | Not on overweight/obesity |
| Garinis ^19^ | 2010 | Intervention (or doses) not approved for obesity |
| Gastaldelli ^20^ | 2009 | Intervention (or doses) not approved for obesity |
| Gastaldelli ^21^ | 2021 | Intervention (or doses) not approved for obesity |
| Goldberg ^22^ | 2025 | Duplicate |
| Golub ^23^ | 2025 | Not RCT |
| Guo ^24^ | 2020 | Intervention (or doses) not approved for obesity |
| Harrison ^25^ | 2009 | Outcomes of interest not reported |
| Harrison ^26^ | 2004 | Not RCT |
| Kaewdech ^27^ | 2024 | No external comparison |
| Kalinowski ^28^ | 2017 | Outcomes of interest not reported |
| Karimi ^29^ | 2025 | Short duration |
| Khoo ^30^ | 2017 | Duplicate |
| Klebanoff ^31^ | 2017 | Not RCT |
| Kuchay ^32^ | 2020 | Not on overweight/obesity |
| Lazo ^33^ | 2010 | Not RCT |
| Lee ^34^ | 2012 | Age< 18 years |
| Lee ^35^ | 2012 | Outcomes of interest not reported |
| Lim ^36^ | 2020 | Not on overweight/obesity |
| Marin-Alejandre ^37^ | 2019 | Duplicate |
| Marin-Alejandre ^38^ | 2021 | No external comparison |
| Mascarò ^39^ | 2022 | Not RCT |
| Montemayor ^40^ | 2022 | No external comparison |
| Montemayor(a) ^41^ | 2022 | Not RCT |
| Nar ^42^ | 2009 | Not RCT |
| Nigam ^43^ | 2014 | Not on overweight/obesity |
| Patel ^44^ | 2015 | Not RCT |
| Rodriguez-Hernandez ^45^ | 2011 | No external comparison |
| Sakane ^46^ | 2020 | Not on MASLD |
| Sherf-Dagan ^47^ | 2018 | Not on overweight/obesity |
| Wei ^48^ | 2023 | No external comparison |
| Wong ^49^ | 2013 | Not on overweight/obesity |
| Wong ^50^ | 2018 | Not on overweight/obesity |
| Yaskolka Mair ^51^ | 2021 | Not on MASLD |
| Zhang ^52^ | 2016 | Duplicate |
| Flint ^53^ | 2021 | Intervention (or doses) not approved for obesity |

# Table 4S. Principal baseline characteristics of the included studies.

| **Study** | **Pub. Year** | **Intervention**  *ID* | **Control**  *Ctrl* | **ID**  *# patients* | **Ctrl**  *# patients* | **Duration**  *weeks* | **BMI at entry**  *kg/m^2^* | **Age at entry**  *years* | **Women**  *%* | **Description of inclusion criteria and PI**  *Liver biopsy* |
| --- | --- | --- | --- | --- | --- | --- | --- | --- | --- | --- |
| *Promrat^54^* | 2010 | MNT | SoC | 21 | 10 | 48 | 33.8 | 48 | 29 | NASH and any fibrosis stage  PI: improvement in NAS^60^, defined as a reduction by at least 3 points or post-treatment NAS of 2 points or less.  Resolution of MASH without worsening of fibrosis was a secondary endpoint. |
| *Loomba^55^* | 2023 | Semaglutide 2.4 mg | Placebo | 47 | 24 | 48 | 35.0 | 59 | 70 | NASH and stage F4 (cirrhosis) or a liver stiffness of greater  than 14 kPa (FibroScan)  PI: improvement in liver fibrosis of one stage or more (using the NASH-CRN fibrosis score^60^) without worsening of NASH.  Resolution of MASH without worsening of fibrosis was a secondary endpoint. |
| *Sanyal^56^* | 2025 | Semaglutide 2.4 | Placebo | 534 | 266 | 72 | 34.6 | 55 | 56 | MASH and stage F2 or F3 (moderate or severe fibrosis)  PI: Resolution of MASH without worsening of fibrosis. |
| *Loomba^57^* | 2024 | Tirzepatide 10-15 mg | Placebo | 95 | 48 | 52 | 36.1 | 54 | 57 | MASH and stage F2 or F3 (moderate or severe fibrosis)  PI: Resolution of MASH without worsening of fibrosis. |
| *Abad^58^* | 2025 | ESG | Placebo | 20 | 20 | 72 | 37.8 | 54 | 45 | MASH and stage F0-F3 (absent to severe fibrosis)  PI: MASH resolution without worsening of fibrosis. |
| *Verrastro^59^* | 2023 | RYGB | SG | 96 | 96 | 52 | 42.1 | 47 | 47 | NASH and stage F0-F3.  PI: Resolution of NASH without worsening of fibrosis. |
|  |  | RYGB | SoC | 96 | 96 |  |  |  |  |  |
|  |  | SG | SoC | 96 | 96 |  |  |  |  |  |

Pub.: Publication; BMI: Body Mass Index; MNT: Medical Nutrition Therapy; SoC: Standard of Care; SG: Sleeve Gastrectomy; ESG: Endosleeve Gastroplasty; RYGB: Roux-en-Y Gastric By-Pass; NASH: Non-Alcoholic Steatohepatitis; MASH: Metabolic dysfunction-associated steatohepatitis; PI: primary endpoint; NAS: NASH Activity Score; CRN: Clinical Research Network.

# Table 5S – Principal characteristics of the network of all available studies on MASH remission without worsening liver fibrosis.

| **Characteristic** | **Value** |
| --- | --- |
| Number of Interventions | 7 |
| Number of Studies | 6 |
| Total Number of Patients in Network | 1,321 |
| Total Possible Pairwise Comparisons | 21 |
| Total Number of Pairwise Comparisons With Direct Data | 7 |
| Is the network connected? | TRUE |
| Number of Two-arm Studies | 5 |
| Number of Multi-Arms Studies | 1 |
| Total Number of Events in Network | 656 |
| Number of Studies With No Zero Events | 6 |
| Number of Studies With At Least One Zero Event | 0 |
| Number of Studies with All Zero Events | 0 |

# Table 6S – NMA league table comparing the effects of all available treatments on MASH remission without worsening liver fibrosis. Treatments are ranked from best to worst along the leading diagonal. Above the leading diagonal are estimates from pairwise meta-analyses, and below the leading diagonal are estimates from network meta-analyses. Relative treatment effects in ranked order for all studies. *SoC: Standard of Care; MNT: Medical Nutrition Therapy; RYGB: Rou-en-Y Gastric By-pass; ESG: Endosleeve Gastroplasty; SG: Sleeve Gastrectomy.*


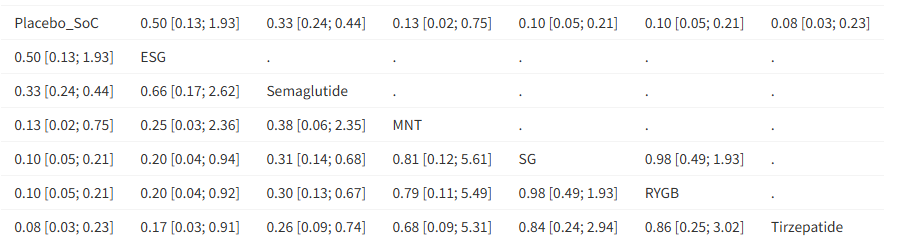


# Table 7S – Assessment of inconsistency across all studies.


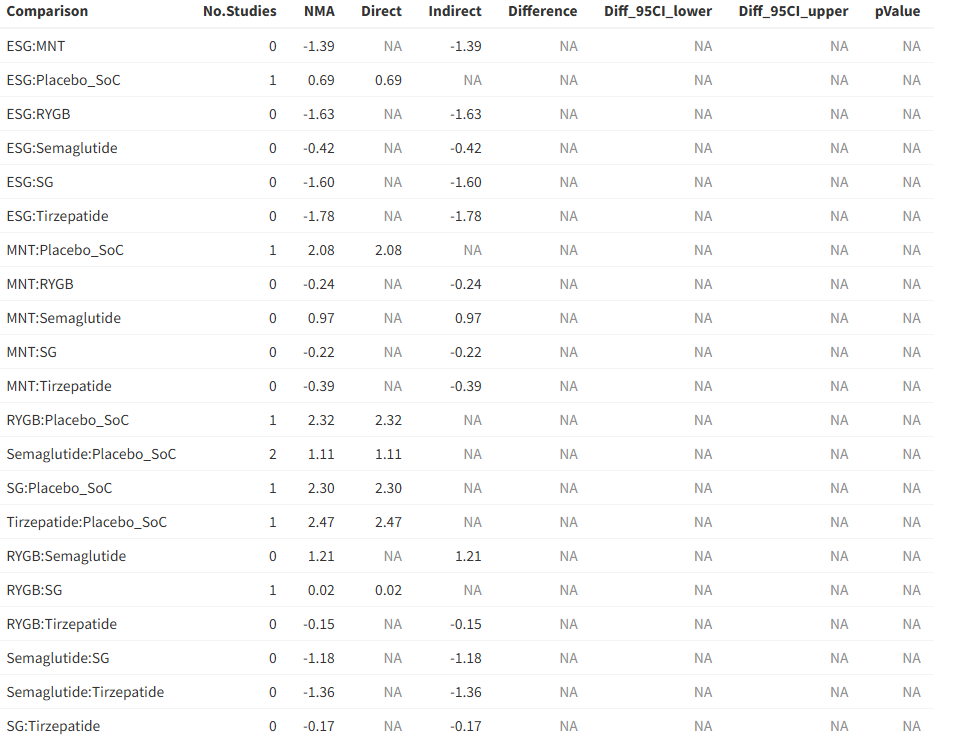


# Table 8S – Evaluation of confidence in the network meta-analysis results considering six domains: within-study bias, reporting bias, indirectness, imprecision, heterogeneity and incoherence. *Domains categories are color-coded as follows: green, low risk of bias; yellow, moderate risk of bias; red, high risk of bias. RYGB: Rou-en-Y Gastric Bypass; SG: Sleeve Gastrectomy; ESG: Endosleeve Gastroplasty; SoC: Standard of Care; MNT: Medical Nutrition Therapy.*


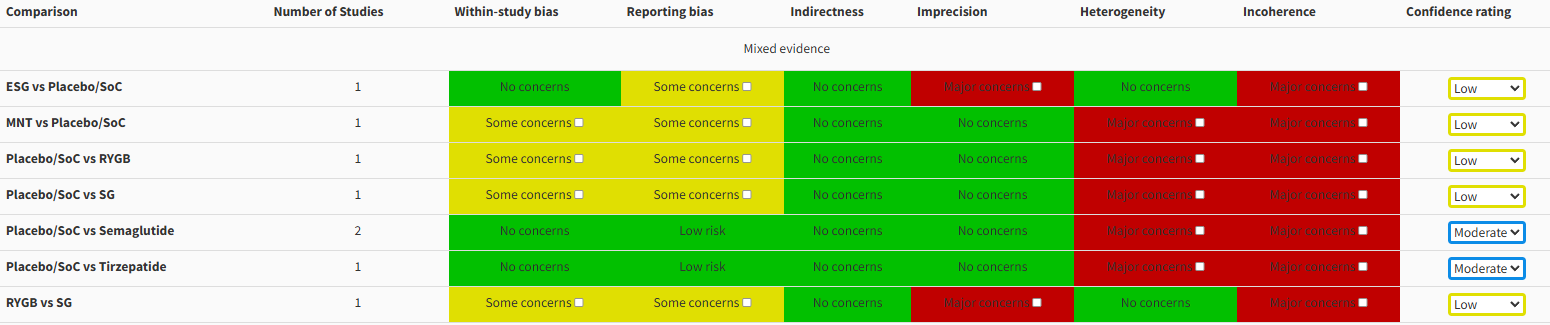


# References

1. Abenavoli L, Greco M, Milic N, Accattato F, Foti D, Gulletta E, et al. Effect of Mediterranean Diet and Antioxidant Formulation in Non-Alcoholic Fatty Liver Disease: A Randomized Study. Nutrients 2017;9(8) (In eng). DOI: 10.3390/nu9080870.

2. Abenavoli L, Greco M, Nazionale I, Peta V, Milic N, Accattato F, et al. Effects of Mediterranean diet supplemented with silybin-vitamin E-phospholipid complex in overweight patients with non-alcoholic fatty liver disease. Expert Rev Gastroenterol Hepatol 2015;9(4):519-27. (In eng). DOI: 10.1586/17474124.2015.1004312.

3. Alkhouri N, Herring R, Kabler H, Kayali Z, Hassanein T, Kohli A, et al. Safety and efficacy of combination therapy with semaglutide, cilofexor and firsocostat in patients with non-alcoholic steatohepatitis: A randomised, open-label phase II trial. J Hepatol 2022;77(3):607-618. (In eng). DOI: 10.1016/j.jhep.2022.04.003.

4. Aller R, de Luis DA, Izaola O, de la Fuente B, Bachiller R. Effect of a high monounsaturated vs high polyunsaturated fat hypocaloric diets in nonalcoholic fatty liver disease. Eur Rev Med Pharmacol Sci 2014;18(7):1041-7. (In eng).

5. Argo CK, Patrie JT, Lackner C, Henry TD, de Lange EE, Weltman AL, et al. Effects of n-3 fish oil on metabolic and histological parameters in NASH: a double-blind, randomized, placebo-controlled trial. J Hepatol 2015;62(1):190-7. (In eng). DOI: 10.1016/j.jhep.2014.08.036.

6. Argo CK, Stine JG, Henry ZH, Lackner C, Patrie JT, Weltman AL, et al. Physical deconditioning is the common denominator in both obese and overweight subjects with nonalcoholic steatohepatitis. Aliment Pharmacol Ther 2018;48(3):290-299. (In eng). DOI: 10.1111/apt.14803.

7. Armstrong MJ, Okanoue T, Sundby Palle M, Sejling AS, Tawfik M, Roden M. Similar weight loss with semaglutide regardless of diabetes and cardiometabolic risk parameters in individuals with metabolic dysfunction-associated steatotic liver disease: Post hoc analysis of three randomised controlled trials. Diabetes, obesity & metabolism 2025;27(2):710-718. (In eng). DOI: 10.1111/dom.16065.

8. Athyros VG, Mikhailidis DP, Didangelos TP, Giouleme OI, Liberopoulos EN, Karagiannis A, et al. Effect of multifactorial treatment on non-alcoholic fatty liver disease in metabolic syndrome: a randomised study. Curr Med Res Opin 2006;22(5):873-83. (In eng). DOI: 10.1185/030079906x104696.

9. Barsalani R, Riesco E, Lavoie JM, Dionne IJ. Effect of exercise training and isoflavones on hepatic steatosis in overweight postmenopausal women. Climacteric 2013;16(1):88-95. (In eng). DOI: 10.3109/13697137.2012.662251.

10. Bomhof MR, Parnell JA, Ramay HR, Crotty P, Rioux KP, Probert CS, et al. Histological improvement of non-alcoholic steatohepatitis with a prebiotic: a pilot clinical trial. Eur J Nutr 2019;58(4):1735-1745. (In eng). DOI: 10.1007/s00394-018-1721-2.

11. Chen Y, Feng R, Yang X, Dai J, Huang M, Ji X, et al. Yogurt improves insulin resistance and liver fat in obese women with nonalcoholic fatty liver disease and metabolic syndrome: a randomized controlled trial. Am J Clin Nutr 2019;109(6):1611-1619. (In eng). DOI: 10.1093/ajcn/nqy358.

12. Craven L, Rahman A, Nair Parvathy S, Beaton M, Silverman J, Qumosani K, et al. Allogenic Fecal Microbiota Transplantation in Patients With Nonalcoholic Fatty Liver Disease Improves Abnormal Small Intestinal Permeability: A Randomized Control Trial. Am J Gastroenterol 2020;115(7):1055-1065. (In eng). DOI: 10.14309/ajg.0000000000000661.

13. Crommen S, Rheinwalt KP, Plamper A, Rösler D, Weinhold L, Metzner C, et al. Prognostic Characteristics of Metabolic Dysfunction-Associated Steatotic Liver in Patients with Obesity Who Undergo One Anastomosis Gastric Bypass Surgery: A Secondary Analysis of Randomized Controlled Trial Data. Nutrients 2024;16(18) (In eng). DOI: 10.3390/nu16183210.

14. de Barros F, Fonseca ABM. Bariatric surgery during the evolution of fatty liver-A randomized clinical trial comparing gastric bypass and sleeve gastrectomy based on transient elastography. Clin Obes 2020;10(6):e12393. (In eng). DOI: 10.1111/cob.12393.

15. Deibert P, Lazaro A, Schaffner D, Berg A, Koenig D, Kreisel W, et al. Comprehensive lifestyle intervention vs soy protein-based meal regimen in non-alcoholic steatohepatitis. World J Gastroenterol 2019;25(9):1116-1131. (In eng). DOI: 10.3748/wjg.v25.i9.1116.

16. Deshmukh A, Sood V, Lal BB, Khanna R, Alam S, Sarin SK. Effect of Indo-Mediterranean diet versus calorie-restricted diet in children with non-alcoholic fatty liver disease: A pilot randomized control trial. Pediatr Obes 2024;19(11):e13163. (In eng). DOI: 10.1111/ijpo.13163.

17. Dichtel LE. The Glucagon-Like Peptide-1 Receptor Agonist, Semaglutide, for the Treatment of Nonalcoholic Steatohepatitis. Hepatology 2021;74(4):2290-2292. (In eng). DOI: 10.1002/hep.31886.

18. Dong F, Zhang Y, Huang Y, Wang Y, Zhang G, Hu X, et al. Long-term lifestyle interventions in middle-aged and elderly men with nonalcoholic fatty liver disease: a randomized controlled trial. Sci Rep 2016;6:36783. (In eng). DOI: 10.1038/srep36783.

19. Garinis GA, Fruci B, Mazza A, De Siena M, Abenavoli S, Gulletta E, et al. Metformin versus dietary treatment in nonalcoholic hepatic steatosis: a randomized study. Int J Obes (Lond) 2010;34(8):1255-64. (In eng). DOI: 10.1038/ijo.2010.40.

20. Gastaldelli A, Harrison SA, Belfort-Aguilar R, Hardies LJ, Balas B, Schenker S, et al. Importance of changes in adipose tissue insulin resistance to histological response during thiazolidinedione treatment of patients with nonalcoholic steatohepatitis. Hepatology 2009;50(4):1087-93. (In eng). DOI: 10.1002/hep.23116.

21. Gastaldelli A, Sabatini S, Carli F, Gaggini M, Bril F, Belfort-DeAguiar R, et al. PPAR-γ-induced changes in visceral fat and adiponectin levels are associated with improvement of steatohepatitis in patients with NASH. Liver Int 2021;41(11):2659-2670. (In eng). DOI: 10.1111/liv.15005.

22. Goldberg DT, Yaskolka Meir A, Tsaban G, Rinott E, Kaplan A, Zelicha H, et al. Novel proteomic signatures may indicate MRI-assessed intrahepatic fat state and changes: The DIRECT PLUS clinical trial. Hepatology 2025;81(1):198-211. (In eng). DOI: 10.1097/hep.0000000000000867.

23. Golub IS, Manubolu VS, Aldana-Bitar J, Dahal S, Verghese D, Alalawi L, et al. The impact of semaglutide on liver fat assessed by serial cardiac CT scans in patients with type 2 diabetes: Results from STOP trial. Nutrition, metabolism, and cardiovascular diseases : NMCD 2025;35(9):104036. (In eng). DOI: 10.1016/j.numecd.2025.104036.

24. Guo W, Tian W, Lin L, Xu X. Liraglutide or insulin glargine treatments improves hepatic fat in obese patients with type 2 diabetes and nonalcoholic fatty liver disease in twenty-six weeks: A randomized placebo-controlled trial. Diabetes research and clinical practice 2020;170:108487. (In eng). DOI: 10.1016/j.diabres.2020.108487.

25. Harrison SA, Fecht W, Brunt EM, Neuschwander-Tetri BA. Orlistat for overweight subjects with nonalcoholic steatohepatitis: A randomized, prospective trial. Hepatology 2009;49(1):80-6. (In eng). DOI: 10.1002/hep.22575.

26. Harrison SA, Fincke C, Helinski D, Torgerson S, Hayashi P. A pilot study of orlistat treatment in obese, non-alcoholic steatohepatitis patients. Aliment Pharmacol Ther 2004;20(6):623-8. (In eng). DOI: 10.1111/j.1365-2036.2004.02153.x.

27. Kaewdech A, Assawasuwannakit S, Churuangsuk C, Chamroonkul N, Sripongpun P. Effect of smartphone-assisted lifestyle intervention in MASLD patients: a randomized controlled trial. Sci Rep 2024;14(1):13961. (In eng). DOI: 10.1038/s41598-024-64988-4.

28. Kalinowski P, Paluszkiewicz R, Ziarkiewicz-Wróblewska B, Wróblewski T, Remiszewski P, Grodzicki M, et al. Liver Function in Patients With Nonalcoholic Fatty Liver Disease Randomized to Roux-en-Y Gastric Bypass Versus Sleeve Gastrectomy: A Secondary Analysis of a Randomized Clinical Trial. Ann Surg 2017;266(5):738-745. (In eng). DOI: 10.1097/sla.0000000000002397.

29. Karimi M, Akhgarjand C, Houjaghani H, Nejad MM, Sohrabpour AA, Poustchi H, et al. The Effect of Intermittent Fasting Diet in Comparison With Low-Calorie Diet on Inflammation, Lipid Profile, Glycemic Index, Liver Fibrosis in Patients With Metabolic-Associated Fatty Liver Disease (MAFLD): A Randomized Controlled Trial. Clin Ther 2025;47(4):e9-e16. (In eng). DOI: 10.1016/j.clinthera.2025.01.007.

30. Khoo J, Hsiang J, Taneja R, Law NM, Ang TL. Comparative effects of liraglutide 3 mg vs structured lifestyle modification on body weight, liver fat and liver function in obese patients with non-alcoholic fatty liver disease: A pilot randomized trial. Diabetes, obesity & metabolism 2017;19(12):1814-1817. (In eng). DOI: 10.1111/dom.13007.

31. Klebanoff MJ, Corey KE, Chhatwal J, Kaplan LM, Chung RT, Hur C. Bariatric surgery for nonalcoholic steatohepatitis: A clinical and cost-effectiveness analysis. Hepatology 2017;65(4):1156-1164. (In eng). DOI: 10.1002/hep.28958.

32. Kuchay MS, Krishan S, Mishra SK, Choudhary NS, Singh MK, Wasir JS, et al. Effect of dulaglutide on liver fat in patients with type 2 diabetes and NAFLD: randomised controlled trial (D-LIFT trial). Diabetologia 2020;63(11):2434-2445. (In eng). DOI: 10.1007/s00125-020-05265-7.

33. Lazo M, Solga SF, Horska A, Bonekamp S, Diehl AM, Brancati FL, et al. Effect of a 12-month intensive lifestyle intervention on hepatic steatosis in adults with type 2 diabetes. Diabetes Care 2010;33(10):2156-63. (In eng). DOI: 10.2337/dc10-0856.

34. Lee S, Bacha F, Hannon T, Kuk JL, Boesch C, Arslanian S. Effects of aerobic versus resistance exercise without caloric restriction on abdominal fat, intrahepatic lipid, and insulin sensitivity in obese adolescent boys: a randomized, controlled trial. Diabetes 2012;61(11):2787-95. (In eng). DOI: 10.2337/db12-0214.

35. Lee YM, Low HC, Lim LG, Dan YY, Aung MO, Cheng CL, et al. Intragastric balloon significantly improves nonalcoholic fatty liver disease activity score in obese patients with nonalcoholic steatohepatitis: a pilot study. Gastrointest Endosc 2012;76(4):756-60. (In eng). DOI: 10.1016/j.gie.2012.05.023.

36. Lim SL, Johal J, Ong KW, Han CY, Chan YH, Lee YM, et al. Lifestyle Intervention Enabled by Mobile Technology on Weight Loss in Patients With Nonalcoholic Fatty Liver Disease: Randomized Controlled Trial. JMIR Mhealth Uhealth 2020;8(4):e14802. (In eng). DOI: 10.2196/14802.

37. Marin-Alejandre BA, Abete I, Cantero I, Monreal JI, Elorz M, Herrero JI, et al. The Metabolic and Hepatic Impact of Two Personalized Dietary Strategies in Subjects with Obesity and Nonalcoholic Fatty Liver Disease: The Fatty Liver in Obesity (FLiO) Randomized Controlled Trial. Nutrients 2019;11(10) (In eng). DOI: 10.3390/nu11102543.

38. Marin-Alejandre BA, Cantero I, Perez-Diaz-Del-Campo N, Monreal JI, Elorz M, Herrero JI, et al. Effects of two personalized dietary strategies during a 2-year intervention in subjects with nonalcoholic fatty liver disease: A randomized trial. Liver Int 2021;41(7):1532-1544. (In eng). DOI: 10.1111/liv.14818.

39. Mascaró CM, Bouzas C, Montemayor S, Casares M, Llompart I, Ugarriza L, et al. Effect of a Six-Month Lifestyle Intervention on the Physical Activity and Fitness Status of Adults with NAFLD and Metabolic Syndrome. Nutrients 2022;14(9) (In eng). DOI: 10.3390/nu14091813.

40. Montemayor S, Bouzas C, Mascaró CM, Casares M, Llompart I, Abete I, et al. Effect of Dietary and Lifestyle Interventions on the Amelioration of NAFLD in Patients with Metabolic Syndrome: The FLIPAN Study. Nutrients 2022;14(11) (In eng). DOI: 10.3390/nu14112223.

41. Montemayor S, Mascaró CM, Ugarriza L, Casares M, Llompart I, Abete I, et al. Adherence to Mediterranean Diet and NAFLD in Patients with Metabolic Syndrome: The FLIPAN Study. Nutrients 2022;14(15) (In eng). DOI: 10.3390/nu14153186.

42. Nar A, Gedik O. The effect of metformin on leptin in obese patients with type 2 diabetes mellitus and nonalcoholic fatty liver disease. Acta Diabetol 2009;46(2):113-8. (In eng). DOI: 10.1007/s00592-008-0067-2.

43. Nigam P, Bhatt S, Misra A, Chadha DS, Vaidya M, Dasgupta J, et al. Effect of a 6-month intervention with cooking oils containing a high concentration of monounsaturated fatty acids (olive and canola oils) compared with control oil in male Asian Indians with nonalcoholic fatty liver disease. Diabetes Technol Ther 2014;16(4):255-61. (In eng). DOI: 10.1089/dia.2013.0178.

44. Patel NS, Doycheva I, Peterson MR, Hooker J, Kisselva T, Schnabl B, et al. Effect of weight loss on magnetic resonance imaging estimation of liver fat and volume in patients with nonalcoholic steatohepatitis. Clin Gastroenterol Hepatol 2015;13(3):561-568.e1. (In eng). DOI: 10.1016/j.cgh.2014.08.039.

45. Rodríguez-Hernández H, Cervantes-Huerta M, Rodríguez-Moran M, Guerrero-Romero F. Decrease of aminotransferase levels in obese women is related to body weight reduction, irrespective of type of diet. Ann Hepatol 2011;10(4):486-92. (In eng).

46. Sakane N, Kotani K, Suganuma A, Takahashi K, Sato J, Suzuki S, et al. Effects of obesity, metabolic syndrome, and non-alcoholic or alcoholic elevated liver enzymes on incidence of diabetes following lifestyle intervention: A subanalysis of the J-DOIT1. J Occup Health 2020;62(1):e12109. (In eng). DOI: 10.1002/1348-9585.12109.

47. Sherf-Dagan S, Zelber-Sagi S, Zilberman-Schapira G, Webb M, Buch A, Keidar A, et al. Probiotics administration following sleeve gastrectomy surgery: a randomized double-blind trial. Int J Obes (Lond) 2018;42(2):147-155. (In eng). DOI: 10.1038/ijo.2017.210.

48. Wei X, Lin B, Huang Y, Yang S, Huang C, Shi L, et al. Effects of Time-Restricted Eating on Nonalcoholic Fatty Liver Disease: The TREATY-FLD Randomized Clinical Trial. JAMA Netw Open 2023;6(3):e233513. (In eng). DOI: 10.1001/jamanetworkopen.2023.3513.

49. Wong VW, Chan RS, Wong GL, Cheung BH, Chu WC, Yeung DK, et al. Community-based lifestyle modification programme for non-alcoholic fatty liver disease: a randomized controlled trial. J Hepatol 2013;59(3):536-42. (In eng). DOI: 10.1016/j.jhep.2013.04.013.

50. Wong VW, Wong GL, Chan RS, Shu SS, Cheung BH, Li LS, et al. Beneficial effects of lifestyle intervention in non-obese patients with non-alcoholic fatty liver disease. J Hepatol 2018;69(6):1349-1356. (In eng). DOI: 10.1016/j.jhep.2018.08.011.

51. Yaskolka Meir A, Keller M, Bernhart SH, Rinott E, Tsaban G, Zelicha H, et al. Lifestyle weight-loss intervention may attenuate methylation aging: the CENTRAL MRI randomized controlled trial. Clin Epigenetics 2021;13(1):48. (In eng). DOI: 10.1186/s13148-021-01038-0.

52. Zhang HJ, He J, Pan LL, Ma ZM, Han CK, Chen CS, et al. Effects of Moderate and Vigorous Exercise on Nonalcoholic Fatty Liver Disease: A Randomized Clinical Trial. JAMA Intern Med 2016;176(8):1074-82. (In eng). DOI: 10.1001/jamainternmed.2016.3202.

53. Flint A, Andersen G, Hockings P, Johansson L, Morsing A, Sundby Palle M, et al. Randomised clinical trial: semaglutide versus placebo reduced liver steatosis but not liver stiffness in subjects with non-alcoholic fatty liver disease assessed by magnetic resonance imaging. Aliment Pharmacol Ther 2021;54(9):1150-1161. (In eng). DOI: 10.1111/apt.16608.

54. Promrat K, Kleiner DE, Niemeier HM, Jackvony E, Kearns M, Wands JR, et al. Randomized controlled trial testing the effects of weight loss on nonalcoholic steatohepatitis. Hepatology 2010;51(1):121-9. (In eng). DOI: 10.1002/hep.23276.

55. Loomba R, Abdelmalek MF, Armstrong MJ, Jara M, Kjær MS, Krarup N, et al. Semaglutide 2·4 mg once weekly in patients with non-alcoholic steatohepatitis-related cirrhosis: a randomised, placebo-controlled phase 2 trial. Lancet Gastroenterol Hepatol 2023;8(6):511-522. (In eng). DOI: 10.1016/s2468-1253(23)00068-7.

56. Sanyal AJ, Newsome PN, Kliers I, Østergaard LH, Long MT, Kjær MS, et al. Phase 3 Trial of Semaglutide in Metabolic Dysfunction-Associated Steatohepatitis. N Engl J Med 2025;392(21):2089-2099. (In eng). DOI: 10.1056/NEJMoa2413258.

57. Loomba R, Hartman ML, Lawitz EJ, Vuppalanchi R, Boursier J, Bugianesi E, et al. Tirzepatide for Metabolic Dysfunction-Associated Steatohepatitis with Liver Fibrosis. N Engl J Med 2024;391(4):299-310. (In eng). DOI: 10.1056/NEJMoa2401943.

58. Abad J, Llop E, Arias-Loste MT, Burgos-Santamaría D, Martínez Porras JL, Iruzubieta P, et al. Endoscopic Sleeve Gastroplasty Plus Lifestyle Intervention in Patients With Metabolic Dysfunction-associated Steatohepatitis: A Multicenter, Sham-controlled, Randomized Trial. Clin Gastroenterol Hepatol 2025;23(9):1556-1566.e3. (In eng). DOI: 10.1016/j.cgh.2024.10.027.

59. Verrastro O, Panunzi S, Castagneto-Gissey L, De Gaetano A, Lembo E, Capristo E, et al. Bariatric-metabolic surgery versus lifestyle intervention plus best medical care in non-alcoholic steatohepatitis (BRAVES): a multicentre, open-label, randomised trial. Lancet 2023;401(10390):1786-1797. (In eng). DOI: 10.1016/s0140-6736(23)00634-7.

60. Kleiner DE, Brunt EM, Van Natta M, Behling C, Contos MJ, Cummings OW, et al. Design and validation of a histological scoring system for nonalcoholic fatty liver disease. Hepatology. 2005;41(6):1313–1321.
